# Genome-Wide Analysis of *WRKY* Genes and Their Response to Hormone and Mechanic Stresses in Carrot

**DOI:** 10.3389/fgene.2019.00363

**Published:** 2019-05-01

**Authors:** Hong Nan, Li-zhi Gao

**Affiliations:** ^1^Plant Germplasm and Genomics Center, Germplasm Bank of Wild Species in Southwest China, Kunming Institute of Botany, Chinese Academy of Sciences, Kunming, China; ^2^University of Chinese Academy of Sciences, Beijing, China; ^3^Institution of Genomics and Bioinformatics, South China Agricultural University, Guangzhou, China

**Keywords:** WRKY transcription factor, carrot, whole genome duplication, expression divergence, hormone and mechanic stresses

## Abstract

The *WRKY* gene family plays a vital role in plant development and environment response. Although previous studies suggested that the *WRKY* genes in carrot (Kuroda type) involved in biotic and abiotic stress responses, the information of *WRKY* genes in the latest version of the carrot genome (*Daucus carota* v2.0, Nantes type carrot) and their response to hormone and injury stresses have not been reported. In this study, we performed a genome-wide analysis of *WRKY*s using a chromosome-scale genome assembly of carrot (*Daucus carota* subsp. *sativus* L.). We identified a total of 67 *WRKY* genes, which were further classified into the three groups. These *WRKY* genes are unevenly distributed on carrot chromosomes. We found that more than half of them were derived from whole-genome duplication (WGD) events, suggesting that WGDs have played a major role during the evolution of the *WRKY* gene family. We experimentally ascertained the expression divergence existed between WGD-derived *WRKY* duplicated gene pairs, which is indicative of functional differentiation between duplicated genes. Our analysis of *cis*-acting elements indicated that *WRKY* genes were transcriptionally regulated upon hormone and mechanic injury stresses. Gene expression analyses by qRT-PCR further presented that *WRKY* genes were involved in hormone and mechanic injury stresses.

## Introduction

Plants often encounter numerous environmental fluctuations containing abiotic and biotic stresses. However, evolutionary alterations have helped them to adapt to these adverse conditions by controlling a network of certain genes through modulating specific transcription factors (TFs). TFs are proteins that can regulate gene expression through binding to specific DNA region adjacent to genes and involve in controlling many important biological processes in the gene transcription regulatory network. In plants, a large number of genes were identified as TFs (Broun, 2004; Yao et al., 2015).

The WRKY family is one of the largest 10 gene families of TFs in higher plants, which has been extensively analyzed in numerous plants since the first *WRKY* gene was identified in *Ipomoea batatas* (Ishiguro and Nakamura, 1994; Rushton et al., 2010). The domain of WRKY transcription factor is about 60 residues in length, containing the highly conserved WRKYGQK signature at the N-terminus, and atypical zinc-finger structure at the C-terminus (Eulgem et al., 2000). In a few WRKY proteins, the conserved WRKYGQK amino acid sequences can also be replaced by other various forms, such as WRKYDHK, WRKYDQK, and WRKYGKK (Li M. Y. et al., 2016). And, the zinc-finger structure is either Cx4-5Cx22-23HxH (C2H2) or Cx7Cx23HxC (C2HC). The WRKY TFs can recognize and bind to the W-box promoter *cis*-element with a consensus C/TTGACC/T sequence (Ciolkowski et al., 2008). Previous studies suggested that the *WRKY* gene family can be divided into three groups based on the number of WRKY domains and the type of zinc-finger (Eulgem et al., 2000; Yousfi et al., 2016). Group I members have two WRKY domains and a C2H2 zinc-finger type. Group II contains one WRKY domain and a C2H2 (Cx4-5Cx22-23HxH) zinc-finger motif. On the basis of primary amino acid sequence, group II can be further divided into the five subgroups, namely II-a, II-b, II-c, II-d, and II-e. Proteins from group III also have a single WRKY domain but with a C2HC (Cx7Cx23HxC) motif (Eulgem et al., 2000; Guo et al., 2014). Recent studies further reported that the *WRKY* gene family is more accurately divided into groups I, II (II-a + II-b, II-c, II-d + II-e) and III based on phylogenetic relationships in higher plants (Huang et al., 2015).

WRKY transcription factors play an important role in developmental and physiological processes of plants. For example, the WRKY transcription factor ScWRKY1 isolated from *Solanum chacoense* was found to be strongly and transiently expressed in fertilized ovules at late torpedo-staged embryos, suggesting a specific role during embryogenesis (Lagac and Matton, 2004). *OsWRKY11* in rice was reported to control flowering time and plant height, and the high expression of *OsWRKY11* leads to dwarfism and late flowering for both long-day and short-day conditions (Cai et al., 2014). In addition, *WRKY* genes were also reported to involve in the regulation of diverse biotic and abiotic stresses, such as bacterial (Tao et al., 2009), pathogens (Kim et al., 2008), salinity (Qin et al., 2015), cold (Ramamoorthy et al., 2008), wounding (Wang et al., 2014), heat, and drought (Wu et al., 2009). The transgenic *Arabidopsis thaliana* lines expressing *VqWRKY52* from wild grape displayed strong resistance to powdery mildew and *Pseudomonas syringae* pv. *tomato* DC3000 while compared with wild type plants (Wang et al., 2017). Over-expression of *VlWRKY48* in *Arabidopsis* could increase the tolerance of fungal infection and drought stresses (Zhao et al., 2017). In *Arabidopsis, AtWRKY30* was greatly induced by salt, drought, H_2_O_2_, and mannitol, and overexpression of *AtWRKY30* was found to enhance plant tolerance to salinity stress (Scarpeci et al., 2013). *GmWRKY21*-transgenic *Arabidopsis* plants were involved in cold stress (Zhou et al., 2008). *GhWRKY40*, a cotton *WRKY* gene, was found to play an important role in the wounding- and pathogen-induced responses in transgenic *Nicotiana benthamiana* (Wang et al., 2014). The grape *VlWRKY3* gene was identified to improve the tolerance to salt and drought stresses and resistance to *Golovinomyces cichoracearum* in transgenic *A. thaliana* (Guo et al., 2018). Besides, overexpression of *OsWRKY11* could increase the tolerance to heat and drought stresses in transgenic rice seedlings (Wu et al., 2009).

WRKY members also play essential roles in signal transduction processes with the involvement of hormones, such as abscisic acid (ABA), salicylic acid (SA), gibberellins (GA), methyl jasmonate (MeJA), and brassinosteroid (BR), which participated in plant immune responses and abiotic stresses. *OsWRKY45* was up-regulated by ABA, and overexpression of this gene in *Arabidopsis* resulted in the enhanced resistance to disease, salt and drought stresses (Qiu and Yu, 2009). In *Arabidopsis, WRKY46* was specifically induced by SA, and it coordinated with *WRKY70* and *WRKY53* in basal resistance against pathogen *P. syringae* (Hu et al., 2012). *AtWRKY12* and *AtWRKY13* were involved in the GA signaling regulation of plant flowering time (Li W. et al., 2016). In American ginseng, methyl jasmonate-inducible *PqWRKY1* gene was involved in osmotic stress and triterpene ginsenoside biosynthesis (Sun et al., 2013). Additionally, **three** *Arabidopsi*s WRKY members, *AtWRKY46, AtWRKY54*, and *AtWRKY70*, were involved in both BR-regulated plant growth and drought response (Chen et al., 2017).

Carrot (*Daucus carota* subsp. *sativu*s L.), belonging to the Apiaceae family, is a globally important root crop with great economic values. Its roots contain high quantities of alpha- and beta-carotene, serving as a good source of vitamin K and vitamin B6 (Pinheiro-Santana et al., 1998). Although the *WRKY* family was preliminarily investigated in a draft genome assembly of carrot (Li M. Y. et al., 2016), tissue-specific expression profiling and the abundance of *WRKY* genes under ABA, GA, and mechanic injury treatments have not yet been studied. The generation of high-quality genome of carrot at chromosome level (Iorizzo et al., 2016) provides an unprecedented opportunity to perform a genome-wide identification of WRKY transcription factor (TF) family. In this study, we accurately characterized the number, structure, chromosomal locations, and phylogenetic relationships of WRKY TF family throughout the carrot genome. We also performed a genome-wide identification of stress-related *cis*-elements in promoters of *WRKY* genes. We comprehensively investigated origins and evolution of the duplicated *WRKY* genes and the expression atlas of *WRKY* genes under abiotic stresses across tissues. This study provides an in-depth insight into the evolution and expression of *WRKY* gene family in carrot.

## Materials and Methods

### Identification of Putative WRKY Proteins in Carrot

To comprehensively identify the carrot *WRKY* genes, genome sequences of this plant were downloaded from the Phytozome (https://phytozome.jgi.doe.gov/pz/portal.html#) (Iorizzo et al., 2016), and the WRKY domain (PF03106) was downloaded from Pfam (http://pfam.xfam.org/). All candidate carrot *WRKY* genes were firstly obtained via searching against the genome with PF03106 file using HMMER3.0 software (http://hmmer.janelia.org/) with parameters as “-E 1e-10 -domE 1e-10.” Then, the conserved domain peptides of the initially identified WRKY members were aligned with MUSCLE (Edgar, 2004) to build carrot-specific HMM file for the *WRKY* family, and the file was used for the next HMM searches. Finally, the sequences were confirmed using SMART database (http://smart.embl-heidelberg.de/) (Letunic et al., 2011). After manually removing incorrect and redundant predicted proteins, the *WRKY* gene members were finally identified in carrot.

As a control, the grape protein sequences were downloaded from Phytozome (https://phytozome.jgi.doe.gov/pz/portal.html#), which were analyzed using the same method as described above. The deduced grape *WRKY* genes were named as *VvWRKY1* to *VvWRKY 59* according to Guo et al. (2014). The sequences of *Arabidopsis WRKY* genes were downloaded from TAIR (https://www.arabidopsis.org/).

### Classification of *DcsWRKY* Genes

The protein sequences of DcsWRKY members were aligned using CLUSTAL method implemented in MEGA and divided into different subgroups according to previous studies (Xie et al., 2005; Xu et al., 2016). In brief, Subgroup I contain two WRKY domains with C2H2 or C2HC. Subgroup II contain one WRKY motif with C2H2 motif and can be divided into five subgroups based on sequences variances in zinc-finger motif, including II-a (CX5CPVKKK(L/V)Q), II-b (CX5CPVRKQVQ), II-c (CX4C), and II-d (CX5CPARKHVE), II-e (CX5CPARK(Q/M)V(E/D). Subgroup III also contain one WRKY motif but with C2HC motif.

### Protein Property and Orthologous Identification

To investigate protein properties of the DcsWRKYs, molecular weight (MW) and isoelectric point (PI) were computed using the online ExPASy-ProtParam tool (http://web.expasy.org/protparam/). In order to identify orthologs in *A. thaliana* for each *DcsWRKY* gene, we performed BLASTP to search against the well-categorized *A. thaliana* WRKY sequences with parameters as “E < 1e-15,” and then, the top hit was collected.

### Mapping *WRKY* Genes on Carrot Chromosomes

To locate positions of *DcsWRKY* genes on the carrot chromosomes, MapInspect Software (http://www.softsea.com/download/MapInspect.html) was used to investigate the distribution of the putative DcsWRKY members based on the genome annotation (GFF3) file of carrot. The file was obtained from Phytozome database (https://phytozome.jgi.doe.gov/pz/portal.html#).

### Phylogenetic Analysis of Conserved Motifs

To investigate phylogenetic relationships of DcsWRKYs and assist their classification, carrot WRKY domain regions together with those from *Arabidopsis* and grape were aligned with CLUSTAL software (Larkin et al., 2007). The phylogenetic tree was created using Neighbor-joining (NJ) method implemented with MEGA (Tamura et al., 2011). Bootstrap values were calculated for 1,000 iterations. In order to further examine the evolution of *DcsWRKY* genes, the full-length proteins from all the predicted DcsWRKYs were aligned with CLUSTAL. Hence, a ML (Maximum-Likelihood) phylogenetic tree was constructed. Bootstrap values were also calculated for 1,000 iterations. MEME suite (http://meme-suite.org/tools/meme) (Bailey et al., 2009) was employed to analyze the motifs in each deduced DcsWRKY proteins. And the parameters were set as follows: maximum number of motifs, 10; minimum width, 6; and maximum width, 50.

### Analysis of Stress-Related *Cis*-Elements

The upstream 1.5 kb sequences of *WRKY* genes were extracted with an in-house Perl script, and the *cis*-elements were identified using the PlantCARE database (http://bioinformatics.psb.ugent.be/webtools/plantcare/html/).

### Syntenic Analysis of *DcsWRKY*s and Timing of Duplication Events

For syntenic analysis of *DcsWRKY* genes, MCScanX (Wang et al., 2012) was employed to detect syntenic gene pairs in the carrot genome. All the proteins were compared against themselves using BLASTP (*e*-value, 1e-10 and outfmt 6). The BLASTP tabular file and a simplified *DcsWRKY* gene location file, which contains chromosome name, gene symbol, start location, and end location, were used as an input for MCScanX with default settings to identify syntenic gene pairs, and the gene type were also determined using MCScanX software.

To estimate divergence times of duplicated genes of *DcsWRKY*, the alignment was performed using MUSCLE and synonymous rate (*Ks*), non-synonymous rate (*Ka*), and evolutionary constraint (*Ka*/*Ks*) were calculated using the PAML software (Yang, 2007). Divergence times of all duplicated *DcsWRKY* genes were calculated using the formula *T* = *Ks*/2r according to the substitution rate of 6.5 × 10^−9^ mutations per site per year (Gaut et al., 1996).

### Plant Materials, Growth Conditions, and Stress Treatments

Carrot tissues (leaves, storage roots, and stems) were obtained from a carrot individual of Nantes type grown in the greenhouse. To obtain the expression profiles of *DcsWRKY* genes under hormone treatments, leaves were treated with 300 μM ABA and 100 μM GA. And then, leaves were sampled at 1, 12, 24, and 48 h after the treatments. Carrot leaves sprayed with sterile water were used as a control. The treatment of mechanical damage stress was performed using forceps, and wounded leaf samples were collected at 1, 4, 8, and 12 h after treatments. Leaves without damage treatments collected on different plants were used as a control. All samples were then harvested, frozen immediately in liquid nitrogen, and stored at −80°C for RNA extraction. Three biological replicate samples were contained for each treatment.

### RNA Extraction and Real-Time PCR Experiment

Total RNA was extracted from samples using a modified CTAB method. And then, the SuperScript III Reverse Transcriptase kit (Invitrogen) was used to synthesize the first-strand cDNA with 1 μg of total RNA from each sample. Real-time quantitative reverse transcription polymerase chain reaction (qRT-PCR) was performed on real-time PCR detection system (Bio-Rad). The primer sequences used were designed based on *WRKY* gene sequences using Primer premier 5.0 software. These sequences were subsequently verified using the BLAST tool at NCBI and a dissociation curve was also analyzed after the PCR reaction to check their specificity. And the gene special primers were listed in Supplementary material (Table S1). Each reaction was carried out in a 10 μL volume, consisting of 5 μL SYBR, 3.6 μL ddH2O, 1 μL template cDNA, and 0.2 μL of each primer. The RT-PCR reaction was performed as follows: 95°C for 30 s, followed by 39 cycles at 95°C for 5 s, and 60°C for 30 s. Then 95°C for 10 s and a melting curve (65–95°C, with 0.5°C increments) was produced to confirm the specificity of amplification. The *TUB* gene was selected as an internal standard for normalization and three technical replicates were completed for each sample (Li M. Y. et al., 2016). The expression levels of leaves at 0 h were separately used as controls (expression = 1.0) for hormone and wounding treatments. The expression levels at other time points and in other organs were normalized accordingly. Error bars show the standard errors for three independent replicates. The 2^−ΔΔ*CT*^ methods were used to analyze relative transcript abundances (Livak and Schmittgen, 2001). All data were expressed as the mean ± SE after normalization of three independent experiments. One-way ANOVA test was employed using SPSS software (version 18.0) to calculate levels of significance. Statistically significant differences were assessed using LSD (Fisher's least significant difference) test, and *P* < 0.05 was adopted as the criterion for statistical significance.

## Results

### Identification of the *WRKY* Genes in Carrot

We predicted 67 non-redundant *WRKY* genes from the carrot genome V2.0 (*DcsWRKY*) using HMM program with open reading frames (ORFs) ranging from 115 to 858 bp. The *DcsWRKY*s were named as *DcsWRKY1* to *DcsWRKY67* according to the order of gene IDs (Table 1). As shown in Table 1, the highly conserved domain WRKYGQK was present in 61 *DcsWRKY* members, whereas the remaining six members contained WRKYGKK, WRKYDQK, and WRKYDHK domains. Of these, WRKYGKK was the most common domain presented in three of the six variants. Slight variations of WRKYGQK domain were also identified in many other plant species, such as broomcorn millet (Yue et al., 2016), cabbage (*Brassica oleracea* var. *captitata*) (Yao et al., 2015), grape (Guo et al., 2014), and tomato (Huang et al., 2012). It has been demonstrated that WRKYGQK domain can bind w-box *cis*-elements to activate the downstream genes. One possible explanation for these WRKY domain variants is an altered binding specificity in the target genes (Guo et al., 2014). For example, *NtWRKY12* in tobacco cannot interact with the w-box motif, but it can bind to wk-box element (van Verk et al., 2008). Therefore, it would be interesting to analyze the function and binding specificities of these six proteins with motif variation. In addition, two genes, *DcsWRKY17* and *DcsWRKY46*, were found to lose the Zinc-finger-like motif of C2HH, but the function of zinc-finger-like motif loss still remains undetermined. Protein properties of each DcsWRKY, including molecular weights and theoretical isoelectric points, were analyzed, which were given in Table 1. The molecular weights ranged from 13.54 (DcsWRKY51) to 94.29 kda (DcsWRKY36), with an average of 40.93 kda. The theoretical isoelectric points varied from 4.82 (DcsWRKY25) to 10.04 (DcsWRKY47). These results indicate a high complexity among the *WRKY* genes in the carrot genome.

**Table 1:** List of the identified *DcsWRKY* genes and their related information.

**Gene name**	**Gene ID**	**ORF (aa)**	**Group**	**pI**	**MW (Kda)**	**Conserved heptapeptide**	**Zinc-finger type**	**Domain number**	**Gene type**	**Ortholog in *A. thaliana***
*DcsWRKY1*	DCAR_000635	293	II-e	5.11	33.48	WRKYGQK	C2HH	1	WGD	*ATWRKY22*
*DcsWRKY2*	DCAR_000809	157	II-c	5.78	17.81	**WRKYGKK**	C2HH	1	Dispersed	*ATWRKY50*
*DcsWRKY3*	DCAR_001382	249	II-c	8.94	28.40	WRKYGQK	C2HH	1	Dispersed	*ATWRKY13*
*DcsWRKY4*	DCAR_004233	567	II-b	6.50	61.36	WRKYGQK	C2HH	1	WGD	*ATWRKY6*
*DcsWRKY5*	DCAR_004610	294	II-e	5.17	32.98	WRKYGQK	C2HH	1	WGD	*ATWRKY22*
*DcsWRKY6*	DCAR_005034	336	III	5.62	37.61	WRKYGQK	C2HC	1	WGD	*ATWRKY41*
*DcsWRKY7*	DCAR_005083	305	II-c	6.86	34.26	WRKYGQK	C2HH	1	Dispersed	*ATWRKY71*
*DcsWRKY8*	DCAR_005143	269	II-e	5.06	30.06	WRKYGQK	C2HH	1	WGD	*ATWRKY65*
*DcsWRKY9*	DCAR_005379	338	III	5.02	38.44	WRKYGQK	C2HC	1	Dispersed	*ATWRKY41*
*DcsWRKY10*	DCAR_005574	306	II-e	5.01	34.50	WRKYGQK	C2HH	1	WGD	*ATWRKY22*
*DcsWRKY11*	DCAR_005640	397	II-e	6.21	43.92	WRKYGQK	C2HH	1	Dispersed	*ATWRKY14*
*DcsWRKY12*	DCAR_005753	282	II-e	4.86	32.51	WRKYGQK	C2HH	1	WGD	*ATWRKY69*
*DcsWRKY13*	DCAR_005872	347	II-d	9.69	38.69	WRKYGQK	C2HH	1	WGD	*ATWRKY21*
*DcsWRKY14*	DCAR_006949	235	II-e	5.38	25.72	WRKYGQK	C2HH	1	Dispersed	*ATWRKY22*
*DcsWRKY15*	DCAR_007136	500	II-b	5.98	54.37	WRKYGQK	C2HH	1	WGD	*ATWRKY61*
*DcsWRKY16*	DCAR_007177	431	I	6.43	47.54	WRKYGQK WRKYGQK	C2H2	2	Dispersed	*ATWRKY4*
*DcsWRKY17*	DCAR_007343	370	II-d	9.53	41.40	WRKYGQK	**Lost**	1	WGD	*ATWRKY21*
*DcsWRKY18*	DCAR_007815	442	II-e	5.28	48.56	WRKYGQK	C2HH	1	Dispersed	*ATWRKY35*
*DcsWRKY19*	DCAR_008173	592	II-b	6.95	64.02	WRKYGQK	C2HH	1	WGD	*ATWRKY6*
*DcsWRKY20*	DCAR_008352	223	II-c	8.13	25.25	WRKYGQK	C2HH	1	Dispersed	*ATWRKY24*
*DcsWRKY21*	DCAR_008638	201	II-c	5.52	23.53	WRKYGQK	C2HH	1	Dispersed	*ATWRKY43*
*DcsWRKY22*	DCAR_008655	351	III	5.48	39.16	WRKYGQK	C2HC	1	WGD	*ATWRKY41*
*DcsWRKY23*	DCAR_009610	203	II-c	6.35	23.25	**WRKYGKK**	C2HH	1	WGD	*ATWRKY51*
*DcsWRKY24*	DCAR_010624	373	III	6.35	41.69	WRKYGQK	C2HC	1	Dispersed	*ATWRKY41*
*DcsWRKY25*	DCAR_010654	362	II-e	4.82	41.19	WRKYGQK	C2HH	1	Dispersed	*ATWRKY22*
*DcsWRKY26*	DCAR_010862	324	II-d	9.46	35.65	WRKYGQK	C2HH	1	WGD	*ATWRKY7*
*DcsWRKY27*	DCAR_012337	260	II-e	5.05	30.07	WRKYGQK	C2HH	1	WGD	*ATWRKY69*
*DcsWRKY28*	DCAR_012521	340	II-d	9.61	37.90	WRKYGQK	C2HH	1	Dispersed	*ATWRKY21*
*DcsWRKY29*	DCAR_012791	339	II-d	9.64	37.08	WRKYGQK	C2HH	1	WGD	*ATWRKY7*
*DcsWRKY30*	DCAR_012904	531	I	6.01	58.63	WRKYGQK WRKYGQK	C2H2/C2H2	2	WGD	*ATWRKY1*
*DcsWRKY31*	DCAR_013280	219	II-e	5.70	24.42	WRKYGQK	C2HH	1	WGD	*ATWRKY65*
*DcsWRKY32*	DCAR_014497	184	II-c	9.42	20.67	WRKYGQK	C2HH	1	Dispersed	*ATWRKY75*
*DcsWRKY33*	DCAR_014683	297	II-d	9.65	32.95	**WKKYDQK**	C2HH	1	WGD	*ATWRKY11*
*DcsWRKY34*	DCAR_014957	754	I	9.23	84.02	WRKYGQK WRKYGQK	C2H2/C2H2	2	WGD	*ATWRKY44*
*DcsWRKY35*	DCAR_016302	334	II-a	8.83	37.24	WRKYGQK	C2HH	1	WGD	*ATWRKY40*
*DcsWRKY36*	DCAR_016536	858	II-b	6.01	94.29	WRKYGQK	C2HH	1	WGD	*ATWRKY72*
*DcsWRKY37*	DCAR_016808	632	II-c	5.86	68.33	WRKYGQK	C2HH	1	Dispersed	*ATWRKY34*
*DcsWRKY38*	DCAR_017993	292	II-d	9.51	32.52	**WKKYDQK**	C2HH	1	WGD	*ATWRKY11*
*DcsWRKY39*	DCAR_018041	318	III	6.32	35.69	WRKYGQK	C2HC	1	Dispersed	*ATWRKY70*
*DcsWRKY40*	DCAR_018294	218	II-e	4.85	25.25	WRKYGQK	C2HH	1	WGD	*ATWRKY65*
*DcsWRKY41*	DCAR_019232	516	I	5.39	56.68	WRKYGQK WRKYGQK	C2H2	2	WGD	*ATWRKY20*
*DcsWRKY42*	DCAR_019267	593	II-b	5.37	65.07	WRKYGQK	C2HH	1	WGD	*ATWRKY6*
*DcsWRKY43*	DCAR_019421	313	II-d	9.80	34.26	WRKYGQK	C2HH	1	WGD	*ATWRKY15*
*DcsWRKY44*	DCAR_019422	313	II-d	9.80	34.26	WRKYGQK	C2HH	1	Tandem	*ATWRKY15*
*DcsWRKY45*	DCAR_019758	541	II-b	5.80	58.78	WRKYGQK	C2HH	1	WGD	*ATWRKY6*
*DcsWRKY46*	DCAR_019969	147	II-c	5.53	16.48	**WRKYGKK**	**Lost**	1	WGD	*ATWRKY50*
*DcsWRKY47*	DCAR_020141	254	II-d	10.04	28.13	**WKKYDHK**	C2HH	1	WGD	*ATWRKY11*
*DcsWRKY48*	DCAR_020153	352	III	4.95	39.17	WRKYGQK	C2HC	1	Dispersed	*ATWRKY55*
*DcsWRKY49*	DCAR_021163	519	II-b	5.50	55.93	WRKYGQK	C2HH	1	WGD	*ATWRKY6*
*DcsWRKY50*	DCAR_021299	233	II-e	5.03	26.12	WRKYGQK	C2HH	1	WGD	*ATWRKY65*
*DcsWRKY51*	DCAR_021802	115	II-c	9.42	13.54	WRKYGQK	C2HH	1	Dispersed	*ATWRKY45*
*DcsWRKY52*	DCAR_021820	482	II-b	6.33	53.84	WRKYGQK		1	Dispersed	*ATWRKY9*
*DcsWRKY53*	DCAR_022855	276	II-a	6.40	31.14	WRKYGQK	C2HH	1	Dispersed	*ATWRKY40*
*DcsWRKY54*	DCAR_023855	564	II-c	5.08	61.22	WRKYGQK	C2HH	1	Dispersed	*ATWRKY2*
*DcsWRKY55*	DCAR_023971	350	III	5.94	38.82	WRKYGQK	C2HC	1	Dispersed	*ATWRKY70*
*DcsWRKY56*	DCAR_024663	175	II-c	9.21	20.18	WRKYGQK	C2HH	1	Dispersed	*ATWRKY75*
*DcsWRKY57*	DCAR_025398	503	I	7.24	55.38	WRKYGQK WRKYGQK	C2H2/C2H2	2	Dispersed	*ATWRKY4*
*DcsWRKY58*	DCAR_025562	343	II-a	8.22	37.74	WRKYGQK	C2HH	1	WGD	*ATWRKY40*
*DcsWRKY59*	DCAR_026069	547	II-b	6.43	60.75	WRKYGQK	C2HH	1	WGD	*ATWRKY72*
*DcsWRKY60*	DCAR_026990	464	II-c	5.70	49.99	WRKYGQK	C2HH	1	Dispersed	*ATWRKY23*
*DcsWRKY61*	DCAR_027713	346	III	5.54	39.13	WRKYGQK	C2HC	1	WGD	*ATWRKY41*
*DcsWRKY62*	DCAR_027985	287	II-e	5.38	31.87	WRKYGQK	C2HH	1	WGD	*ATWRKY65*
*DcsWRKY63*	DCAR_028753	294	II-c	4.83	32.74	WRKYGQK	C2HH	1	Dispersed	*ATWRKY49*
*DcsWRKY64*	DCAR_028893	528	I	6.48	58.46	WRKYGQK WRKYGQK	C2H2/C2H2	2	Proximal	*ATWRKY33*
*DcsWRKY65*	DCAR_030029	287	II-c	6.20	31.73	WRKYGQK	C2HH	1	Dispersed	*ATWRKY57*
*DcsWRKY66*	DCAR_030459	287	II-c	6.60	32.04	WRKYGQK	C2HH	1	Dispersed	*ATWRKY48*
*DcsWRKY67*	DCAR_031047	770	II-b	8.28	84.42	WRKYGQK	C2HH	1	WGD	*ATWRKY61*

*Six DcsWRKYs without the conserved WRKYGQK signature and two DcsWRKYs without the Zinc-finger-like motif were bold marked*.

### Classification of *DcsWRKY* Genes

**Figure 1:**
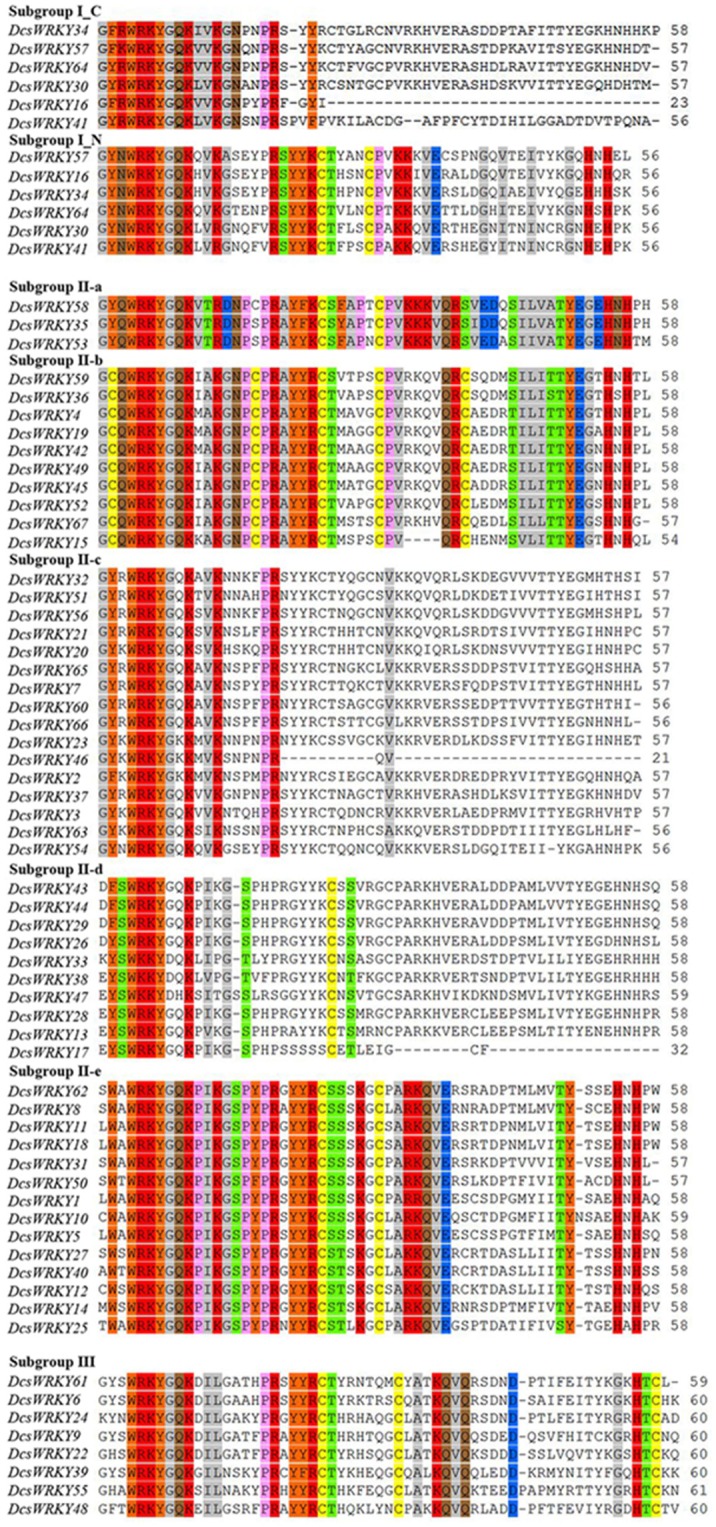
Multiple sequence alignment of the WRKY domain among carrot *WRKY* genes. The alignment was performed by Clustal W. Dashes indicate gaps. “N” and “C” indicate the N-terminal and C-terminal WRKY domain of a specific *WRKY* gene.

**Figure 2:**
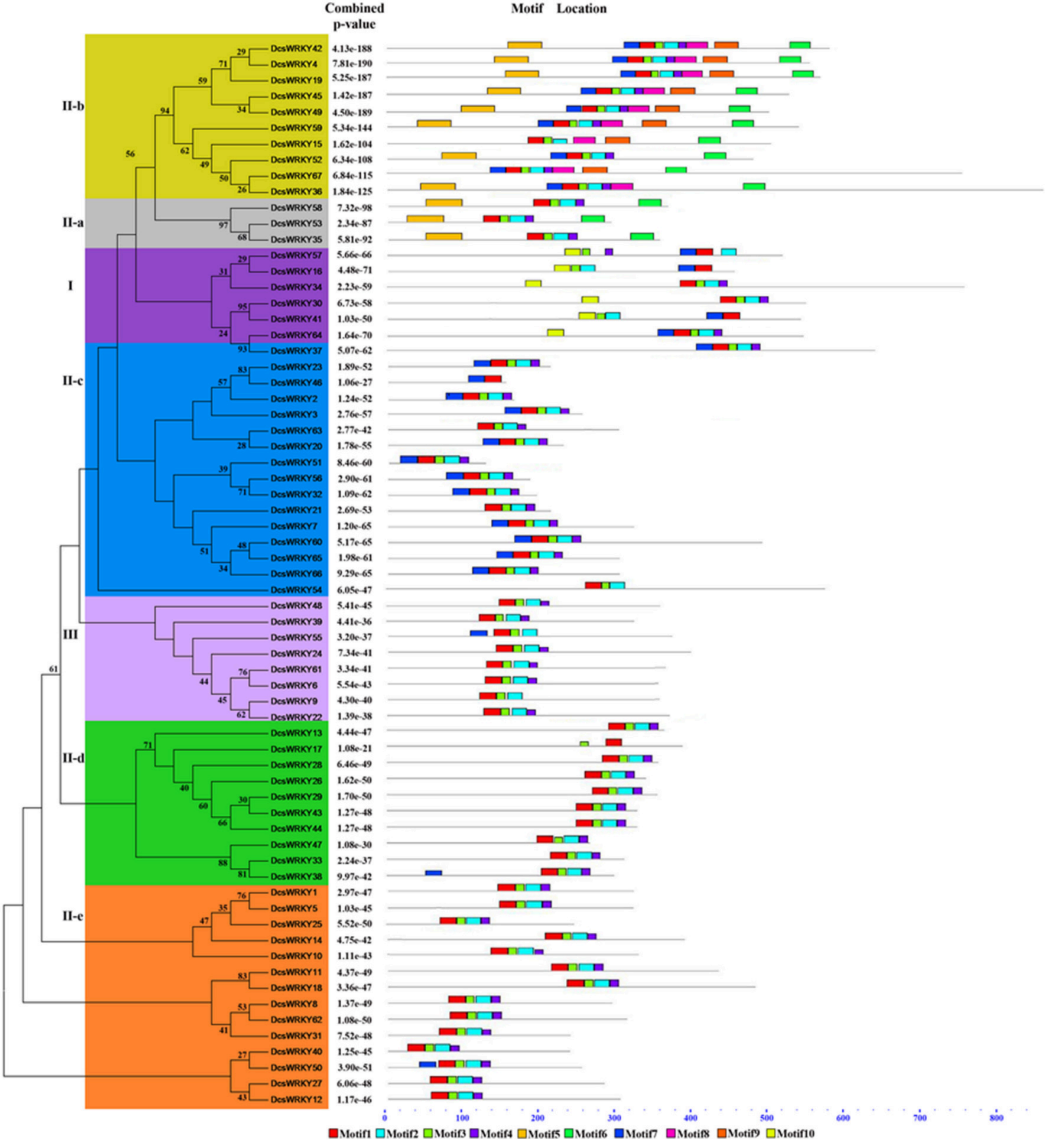
Phylogenetic analysis and conserved motif compositions of the *WRKY* genes in carrot. Phylogenetic tree was constructed using the maximum-likelihood method based on alignments of complete predicted proteins. The conserved motifs were detected using MEME software and represented by colored boxes. Reliability of the predicted tree was tested using bootstrapping with 1,000 replicates. The length of WRKY proteins can be estimated using the scale at the bottom and conserved motifs were shown in Table 2.

Based on the number of WRKY domains and the zinc finger type, the putative WRKY proteins could be divided into three groups, namely, groups I, II, and III (Eulgem et al., 2000). As shown in Table 1 and Figure 1, there were six members in Group I, which contained two WRKYGQK domains, and two zinc-finger motifs of C2H2 type except for *DcsWRKY16* and *DcsWRKY41* without the zinc-finger structure at C-terminus. Group II, in particular, could be further divided into the five subgroups (II-a, II-b, II-c, II-d, II-e) and contained 3, 10, 16, 10, 14 WRKY members, respectively. Eight members of group III were found in the carrot genome with only one WRKYGQK domain and a zinc-finger motif of C2HC type. Finally, two *DcsWRKY* genes (*DcsWRKY17* and *DcsWRKY46*) with incomplete structures were not assigned to any of the subgroups. However, based on the phylogenetic tree of WRKY full-protein (Figure 2), they could be classified into subgroup II-d and II-c, respectively. Detailed information about the type of *DcsWRKY* genes and domains is given in Figure 1. Orthologs are genes in different genomes that originate from a common ancestral gene by speciation and often retain similar functions (Remm et al., 2001), and thus, comparisons between a model species and a less-studied species allow us to understand genomic information of less-studied taxa (Lyons et al., 2008). Orthologs, between *A. thaliana* and carrot, were detected using BLASTP with e value 1e-15. Our results showed that most WRKY members were found to expand nearly two times more than *A. thaliana*.

**Table 2:** Conserved motifs of DcsWRKY proteins.

**Motif ID**	**Conservative motifs**	***E*-value**	**Width**	**Sites**	**Description**
Motif 1	ILDDGYSWRKYGQKPIKGSPY	2.1e−905	21	67	WDP^*^
Motif 2	CPARKQVZRSSEDPSILIT	2.1e−567	19	65	WDP
Motif 3	PRSYYRCTSSK	1.8e−395	11	66	WDP
Motif 4	TYEGEHNHPLP	5.60E−274	11	59	WDP
Motif 5	KBELGALQAELERMNTENKRLRDMLDQVTNNYNTLQTHLVTIMQQQ	4.10E−126	46	11	
Motif 6	SJAAATKAJTSDPNFTAALAAAISSIIGG	9.30E−117	29	13	
Motif 7	KKTEKKVRKPRVAVRTRSEVD	1.10E−94	21	29	
Motif 8	PAAMAMASTTSAAARMLLSGSMSSADGIL	3.40E−83	29	9	
Motif 9	LPPFSSSMATISASAPFPTVTLDLTQSPNPLQY	1.00E−65	33	8	
Motif 10	KPSDDGYNWRKYGQKQVKGSE	1.80E−47	21	6	WDP

* *WDP indicates a part of WRKY domain*.

### Distribution of *DcsWRKY* Genes on Chromosomes

**Figure 3:**
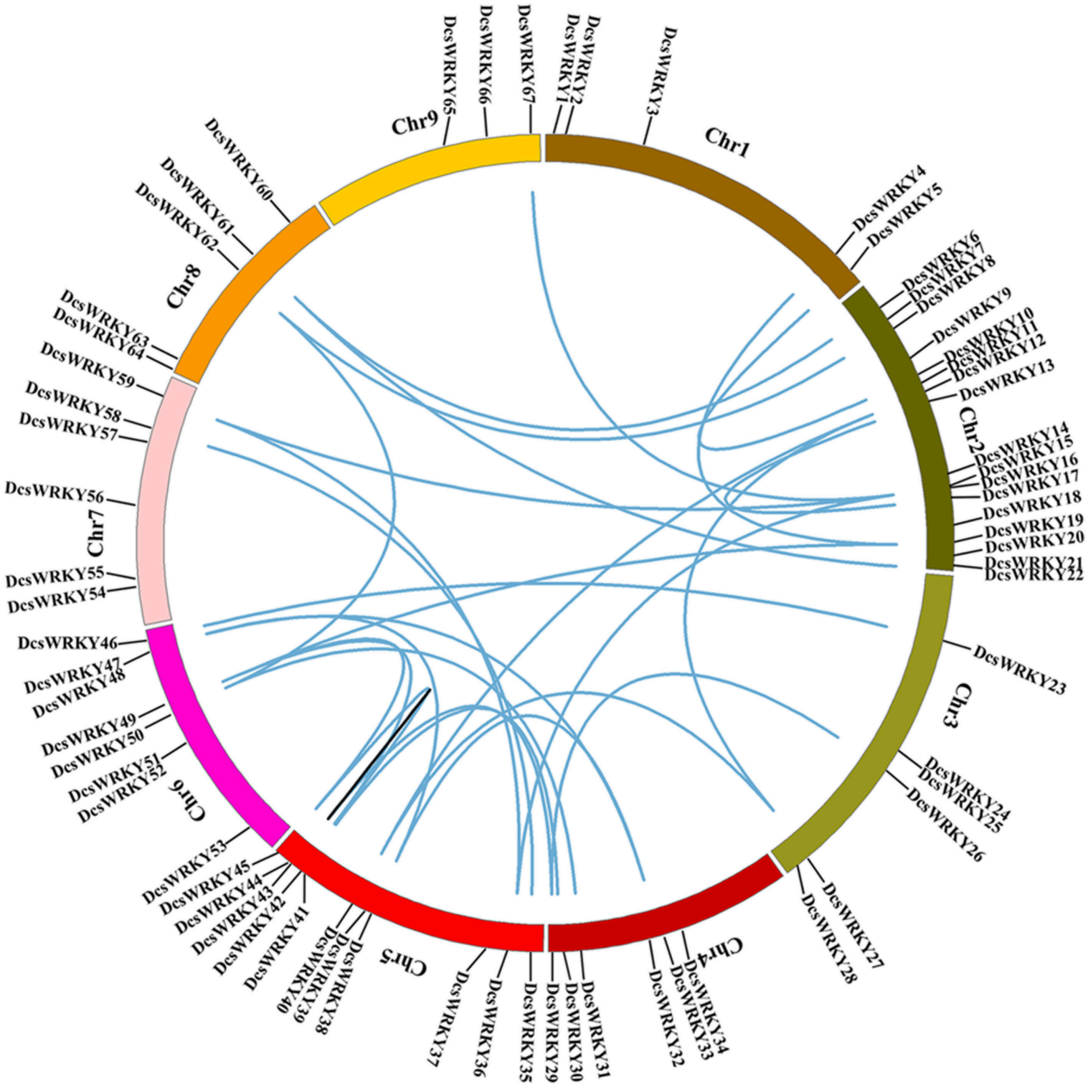
Chromosome distribution and synteny analysis of carrot *WRKY* genes. Chromosomes 1–9 are shown with different colors. The approximate distribution of each gene is marked with a short black line. Blue curves indicate duplicated *DcsWRKY* genes, and black lines in the circle indicate tandem duplicated genes.

The genomic distribution of *DcsWRKY* genes was investigated by positioning their approximate positions on each chromosome. As shown in Figure 3, among the nine chromosomes, *DcsWRKY* genes were unevenly distributed, and the numbers on each chromosome were not related to their sequence lengths (Figure 3, Table S2). Chromosome two harbored the majority of *WRKY* genes (25.37%), including one members in group I, 13 in group II, and three in group III of the *DcsWRKY* gene family, followed by 11 genes on chromosome five (one in group I, nine in group II, and one in group III), while chromosome nine only contained three *DcsWRKY*s (two in group II-c and one in group II-b). Chromosome one, the longest chromosome, only had five in group II of *DcsWRKY* genes.

### Conserved Motifs and Phylogenetic Relationships of the *DcsWRKY* Gene Family

To better characterize the *DcsWRKY* gene family, we predicted the conserved motifs in DcsWRKY proteins by MEME online software (Table 2). An unrooted phylogenetic tree was constructed from the 67 DcsWRKY proteins using ML method (Figure 2). In total, 10 distinct motifs were identified, and all DcsWRKY members could be divided into seven large subgroups (i.e., I, II a-e, and III) previously found in higher plants, which shared similar motifs (Xiong et al., 2013; Wen et al., 2014; Chen et al., 2018). As illustrated in Table 2 and Figure 2, after assessing the distribution of the motifs from these subgroups, half of them were identified lying around the WRKY, such as motifs 5–9, while other motifs 1, 2, 3, 4, and 10 were located in the WRKY domain. Overall, the same subgroup shared similar motif compositions, indicating a highly functional conservation. Subgroups II-a and II-b were closely related, and motifs 5 and 6 were uniquely dispersed across them. In most cases, motifs 5 and 6 appeared as a pair, indicating that they are functionally related to the subgroups. Subgroups II-d and II-e were clustered together and contained motifs 1, 2, 3, and 4 except DcsWRKY17. Of these 10 motifs, all groups shared motifs 1, 2, 3, and 4, which partially represented the distribution of the C-terminal domain. Motif 10 was only found in group I, which partially exemplified the distribution of the N-terminal domains.

**Figure 4:**
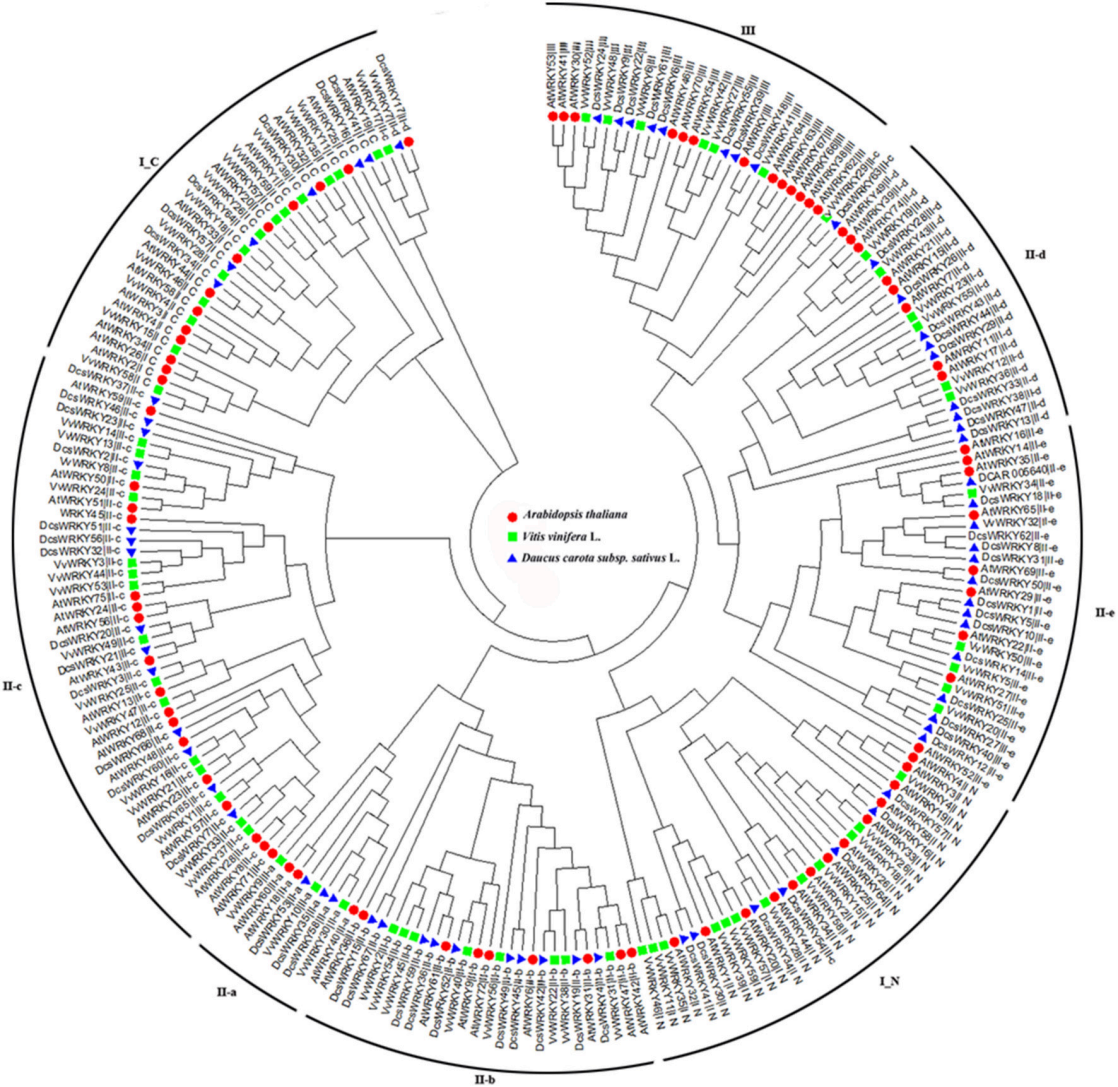
Phylogenetic tree based on WRKY domains from carrot, *Arabidopsis*, and grape domains of the *WRKY* gene family. The phylogenetic tree was constructed using Neighbor-Joining method based on 197 WRKY domain sequences. Reliability of the predicted tree was tested using bootstrapping with 1,000 replicates. The tree was divided into eight phylogenetic subgroups, namely I_N, I_C, II-a–II-e, and III. The *WRKY* genes of *Arabidopsis*, grape, and carrot were denoted by circles, diamonds, and triangles, respectively.

To understand the diversification and evolution of the *DcsWRKY* gene family we compared the *WRKY* members from the two other sequenced plant genomes (grape and *Arabidopsis*). An unrooted phylogenetic tree was constructed using the Neighbor-Joining (NJ) method implemented with MEGA from the 197 conserved WRKY domains among the three plant species. As shown in Figure 4, the complete WRKY domains were divided into eight subgroups (i.e., I_N, I_C, II a-e, and III), and the WRKY members belonging to the same group from these three species similarly had conserved domain compositions. The ancestral groups I_N and I_C were separately grouped on the phylogenetic tree, while I_C was the basal clade of the phylogenetic tree, which is consistent with the hypothesis that the group I was the oldest group (Wu et al., 2005). In addition, I_C and subgroup II-c were closely clustered, and subgroup II-c appeared polyphyletic.

### Stress-Related *Cis*-Elements in Promoters of *DcsWRKY* Genes

To investigate the evolution and functional diversification of the *WRKY* members, we retrieved, and analyzed the upstream 1.5 Kb promoter regions of the *DcsWRKY* genes. A number of *cis*-acting regulatory elements, including 9 elements related to plant development and 16 motifs related to stress responses, were analyzed, and the 25 elements were represented in Table S3. *Cis*-elements related to plant growth and development include light responsive elements (box4, G-box, sp1, and ACE), endosperm expression (skn-1_motif and GCN4_motif), circadian control (circadian), meristem expression (CAT-box), and meristem-specific activation (CCGTCC-box). *Cis*-elements related to stress responses comprise eight hormone responsive elements (CGTCA-motif, TGACG-motif, ABRE, GARE-motif, TATC-box, p-box, TGA-element, and ERE), wound-responsive element (WUN-motif), anaerobic induction element (ARE), low-temperature responsive element (LTR), and so on. As shown in Table S3, each *DcsWRKY* gene contained more than one *cis*-acting regulatory element in their promoter regions, and most *DcsWRKY* genes contained box4, G-box, skn-1, and MBS motif.

### Origin and Evolution of the Duplicated *DcsWRKY* Genes

Regarded as the main evolutionary force in both plants and animals, whole-genome duplication (WGD) has been extensively found in most eudicots, following by extensive gene loss (Sémon and Wolfe, 2007). *D*. *carota* has experienced two recent species-specific WGD events, Dc-α, and Dc-β, following the earlier γ paleohexaploidy event (~117 MYA) shared by core eudicots (Jiao et al., 2012). Note that these two carrot-specific WGDs occurred approximately 43 and 70 million years ago, respectively. To understand the role of WGD events in the carrot *WRKY* genes we performed syntenic analysis using MCScanX. To investigate the collinearity of the *WRKY* gene family, all protein sequences of *D*. *carota* subsp. *sativus* were first identified using BLASTP; the resulting BLASTP hits were then compiled as the input for MCScanX to classify duplicated gene pairs under the default settings. And then, custom Perl scripts were used to collect syntenic gene pairs. According to previous studies (Liu and Ekramoddoullah, 2009; Bi et al., 2016), two or more adjacent homologous genes located on a single chromosome were regarded as tandem duplicated genes, while homologous genes located on different genomics regions or chromosomes were regarded as WGD derived genes or segmentally duplicated genes. The collinear relationships of the duplicated pairs in the *DcsWRKY* gene family were shown in Table 3. Of the 67 *DcsWRKY* genes, 27 pairs of WGD duplicated genes (36 *DcsWRKY* members), and only one pair of tandem duplicated genes (*DcsWRKY43* and *DcsWRK44*) were identified, indicating that the *WRKY* genes were mainly derived from whole genome duplication events, which acted as a major force to drive the evolution of the *DcsWRKY* gene family. In many other plants, most *WRKY* genes were also found to derive from whole genome duplication events, such as cabbage, peanut (Song et al., 2016), and soybean (Yin et al., 2013). As shown in Figure 3, chromosomes two and five had most of WGD-derived *DcsWRKY* duplicated genes, while chromosome nine only had one (*DcsWRKY67*). And, the tandem duplicated gene pairs were located on chromosome five. In addition, the two duplicated genes in one pair belonged to the same WRKY group (Table 3), suggesting that duplicated genes may have a similarly conserved function.

**Table 3:** *Ka*/*Ks* calculation and divergence times of the duplicated *DcsWRKY* gene pairs in syntenic blocks.

**Duplicated gene pairs**		**Group**	***Ka***	***Ks***	***Ka*/*Ks***	**Purify selcetion**	**Duplicated type**	**Time (MYA)**	**Duplication event**
*DcsWRKY4*	*DcsWRKY19*	II-b	0.18	0.98	0.19	Yes	WGD	75.14	Dc-β
*DcsWRKY5*	*DcsWRKY10*	II-e	0.44	1.34	0.33	Yes	WGD	102.70	WGT (γ)
*DcsWRKY13*	*DcsWRKY17*	II-d	0.28	0.56	0.50	Yes	WGD	42.87	Dc-α
*DcsWRKY12*	*DcsWRKY27*	II-e	0.26	0.77	0.33	Yes	WGD	59.61	Dc-β
*DcsWRKY12*	*DcsWRKY40*	II-e	0.21	0.76	0.28	Yes	WGD	58.45	Dc-β
*DcsWRKY15*	*DcsWRKY36*	II-b	0.78	0.93	0.83	Yes	WGD	71.84	Dc-β
*DcsWRKY19*	*DcsWRKY49*	II-b	0.45	1.82	0.25	Yes	WGD	139.63	WGT (γ)
*DcsWRKY15*	*DcsWRKY59*	II-b	0.70	1.47	0.48	Yes	WGD	113.21	WGT (γ)
*DcsWRKY6*	*DcsWRKY61*	III	0.17	0.60	0.28	Yes	WGD	46.54	Dc-α
*DcsWRKY8*	*DcsWRKY62*	II-e	0.19	0.77	0.24	Yes	WGD	59.13	Dc-β
*DcsWRKY22*	*DcsWRKY61*	III	0.58	1.45	0.40	Yes	WGD	111.49	WGT (γ)
*DcsWRKY15*	*DcsWRKY67*	II-b	0.42	0.56	0.74	Yes	WGD	43.12	Dc-α
*DcsWRKY26*	*DcsWRKY29*	II-d	0.40	1.97	0.20	Yes	WGD	151.61	WGT (γ)
*DcsWRKY27*	*DcsWRKY40*	II-e	0.14	0.67	0.20	Yes	WGD	51.76	Dc-α
*DcsWRKY23*	*DcsWRKY46*	II-c	0.28	0.71	0.39	Yes	WGD	54.80	Dc-α
*DcsWRKY33*	*DcsWRKY38*	II-d	0.30	1.21	0.25	Yes	WGD	93.29	WGT (γ)
*DcsWRKY29*	*DcsWRKY43*	II-d	0.28	1.16	0.24	Yes	WGD	88.96	WGT (γ)
*DcsWRKY30*	*DcsWRKY41*	I	0.21	0.45	0.47	Yes	WGD	34.88	Dc-α
*DcsWRKY31*	*DcsWRKY50*	II-e	0.35	1.46	0.24	Yes	WGD	112.49	WGT (γ)
*DcsWRKY33*	*DcsWRKY47*	II-d	0.68	1.70	0.40	Yes	WGD	131.05	WGT (γ)
*DcsWRKY42*	*DcsWRKY45*	II-b	0.29	1.32	0.22	Yes	WGD	101.63	WGT (γ)
*DcsWRKY45*	*DcsWRKY49*	II-b	0.15	0.63	0.23	Yes	WGD	48.84	Dc-α
*DcsWRKY38*	*DcsWRKY47*	II-d	0.81	1.90	0.42	Yes	WGD	146.45	WGT (γ)
*DcsWRKY42*	*DcsWRKY49*	II-b	0.26	1.26	0.21	Yes	WGD	96.63	WGT (γ)
*DcsWRKY36*	*DcsWRKY59*	II-b	0.47	0.87	0.54	Yes	WGD	66.62	Dc-β
*DcsWRKY35*	*DcsWRKY58*	II-a	0.24	1.41	0.17	Yes	WGD	108.08	WGT (γ)
*DcsWRKY50*	*DcsWRKY62*	II-e	0.54	1.83	0.29	Yes	WGD	140.55	WGT (γ)
*DcsWRKY43*	*DcsWRKY44*	II-d	–	–	–	–	Tandem	–	–

*WGT (γ), the ancestral γpaleohexaploidy event shared by core eudicots; Dc-α, carrot-specific WGD events; Dc-β, carrot-specific WGT events; Ks, synonymous substitutions per synonymous site; Ka, non-synonymous substitutions per non-synonymous site*.

We calculated the synonymous (*Ks*), non-synonymous (*Ka*) substitution rate and *Ka*/*Ks* ratios of the duplicated *WRKY* genes in carrot. The *Ka* values ranged from 0.14 to 0.81, and *Ks* varied from 0.45 to 1.97. Previous studies suggested that *Ka*/*Ks* < 1, *Ka*/*Ks* = 1, and *Ka*/*Ks* > 1 indicate purifying selection, neutral evolution, and positive selection, respectively (Tang et al., 2013; Song et al., 2016). Of the 28 duplicated gene pairs, all WGD duplicated *DcsWRKYs* had *Ka*/*Ks* < 1, ranging from 0.17 to 0.83. The results suggested purifying selection act on these duplicated gene pairs, which agreed with what observed in peanuts (Song et al., 2016), and *B. rapa* (Tang et al., 2013).

To time these duplicated *DcsWRKY* genes, the *Ks* values were served as proxies for the divergent events, and divergence dates were calculated using the function: *Ks*/2r. As described in Table 3, divergence dates spanned ~34.88–151.61 million years, suggesting that the duplicated genes in carrot occurred from the gamma polyploidy event. Carrot underwent one γ paleohexaploidy event and two species-specific WGDs, namely Dc-α and Dc-β, which occurred approximately 117, 43, and 70 million years ago, respectively. Thus, there were 14 (~51.9%) duplication events with 22 genes occurred during gamma polyploidy event, while 7 (~25.9%) and 6 (~22.2%) duplication events were identified during Dc-α and Dc-β events, respectively. Moreover, seven *DcsWRKY* members (*DcsWRKY15, DcsWRKY27, DcsWRKY40, DcsWRKY45, DcsWRKY49, DcsWRKY59, DcsWRKY61*, and *DcsWRKY62*) experienced at least two rounds of WGD events, and only the *DcsWRKY15* gene involved in all these three WGD events.

### Expression of *DcsWRKY* Genes Across Different Tissues

**Figure 5:**
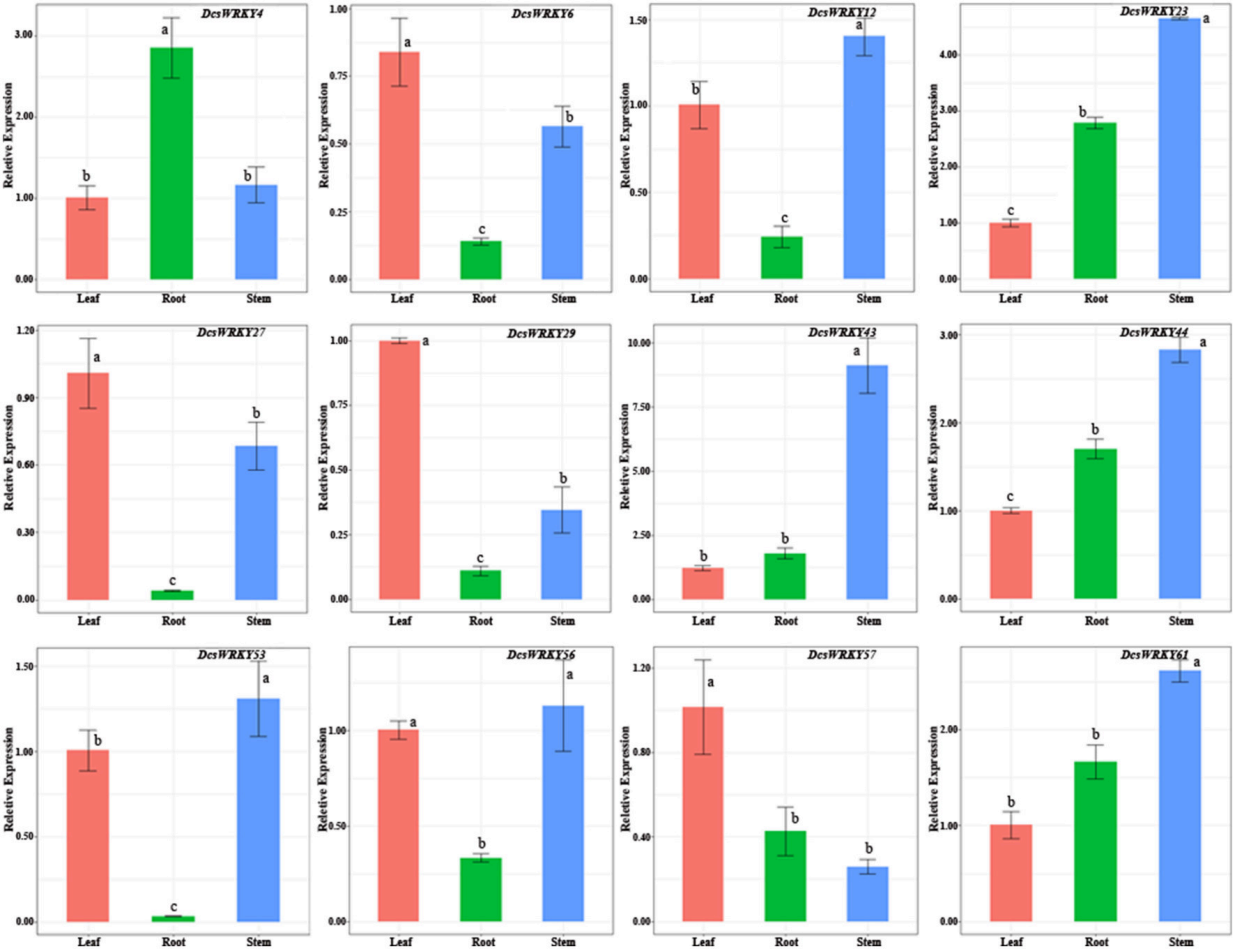
Real-time quantitative PCR expression levels of selected genes in various organs. The y-axis represents the relative expression levels of genes. The x-axis shows different tissues. Error bars represent standard errors of the mean in three biological replicates. One-way ANOVA test (*P* < 0.05, LSD) was used to statistically evaluate the significance among those samples. The histogram bars labeled with different letters (a, b, c, and d) above histogram bars are significantly different (LSD test, *P* < 0.05). LSD designates Fisher's least significant difference test.

To examine patterns and expression levels of *DcsWRKY* genes, 12 carrot *WRKY* genes in three tissues (storage roots, leaves, and stems) were researched through qRT-PCR experiments. Our results showed that *DcsWRKY* genes exhibited distinct expression patterns, and tissue-specific expression of *WRKY* genes was also observed in carrot (Figure 5). For instance, *DcsWRKY29* and *DcsWRKY57* were particularly highly expressed in leaves but levels of expression were low in roots and stems. *DcsWRKY43* exhibited extremely low levels in leaves and roots, whereas it was highly expressed in stems. Previous studies reported that the highly expressed *WRKY* genes in certain tissues were often found to regulate target genes involved in important processes of growth and development (Yu et al., 2012). The three *DcsWRKY* genes (*DcsWRKY4, DcsWRKY56*, and *DcsWRKY57*) showed no significant expression difference between at least two tissues, suggesting that they likely play an ubiquitous role in carrot. Furthermore, *DcsWRKY44* and *DcsWRKY61* were constitutively expressed in each tested tissue and shared a similar expression trend, indicating their putative redundant functions in the development and physiological processes of these tissues.

We also investigated the expression divergence between the duplicated *DcsWRKY* genes. *DcsWRKY6* and *DcsWRKY61* were duplicated gene pairs generated during Dc-α event, which are orthologous to *AtWRKY41* in *Arabidopsis* (Tables 1, 3). As shown in Figure 5, *DcsWRKY6* was quite lowly expressed in stems, while the expression level of *DcsWRK61* was extremely high in stems. Another exemplar pairs of duplicated genes, *DcsWRKY29*, and *DcsWRKY43*, were produced during the γ paleohexaploidy event. *DcsWRKY29* was up-regulated in leaves and down-regulated in stems, while its paralog *DcsWRKY43* was down-regulated in leaves and up-regulated in stems. Previously numerous studies suggested that tissue-specific expression divergence is one of the most important indicators of functional differentiation between duplicated genes, and thus gene duplication plays a key role in the growth of gene networks (Makova and Li, 2003; Li et al., 2005). Thus, the expanded *WRKY* genes might result in novel biological function after gene duplication events, which are beneficial to regulate various physiological processes by removing their redundancy.

### Expression Pattern of *DcsWRKY* Genes Under Stresses

**Figure 6:**
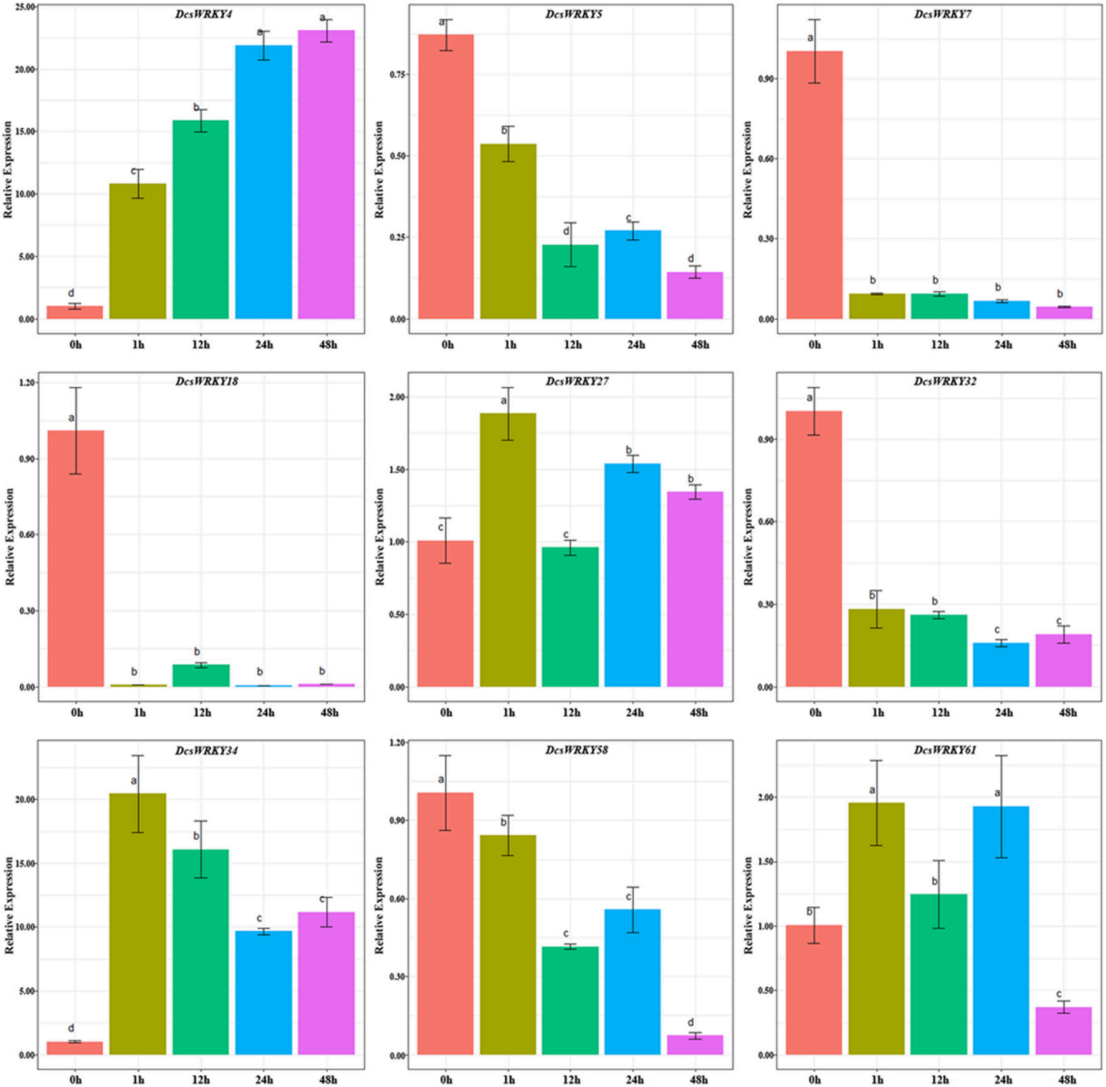
Expression patterns of *DcsWRKY* genes under ABA treatments. Samples collected at 0, 1, 12, 24, and 48 h after treatment. The expression levels were normalized to control samples. Error bars represent standard errors of the mean in three biological replicates. One-way ANOVA test (*P* < 0.05, LSD) was used to statistically evaluate the significance among those samples. The histogram bars labeled with different letters (a, b, c, and d) above histogram bars are significantly different (LSD test, *P* < 0.05). LSD designates Fisher's least significant difference test.

WRKY transcription factors were found to respond to various stresses that may result from the upstream specific *cis*-elements to regulate gene expressions (Shinozaki et al., 2003; Kim and Zhang, 2004). To understand the roles of these *DcsWRKY*s in response to abiotic stresses, we exposed ABA and GA to leaves, and performed mechanic injury treatments in carrot. The expression profiles of *DcsWRKY* genes that contained corresponding *cis*-elements were examined using qRT-PCR experiments. In hormone treatments, a total of nine *DcsWRKY* genes with ARRE motif were selected to examine patterns of gene expression under ABA treatment (Figure 6). Our results showed that *DcsWRKY5, DcsWRKY7, DcsWRKY18*, and *DcsWRKY32* were evidently down-regulated by 2-, 10-, 136-, and 12-fold after 1 h, respectively, whereas *DcsWRKY4* increased by almost 10 times at 1 h and had a maximum expression level at 48 h. Moreover, the expression level of *DcsWRKY58* slightly decreased after 1 h and reached the lowest level after 48 h. We also observed that genes belonging to the same *DcWRKY* subgroup could show a distinct expression trend, such as *DcWRKY5* (II-e), *DcsWRKY18* (II-e), and *DcsWRKY27* (II-e) (Figure 6).

**Figure 7:**
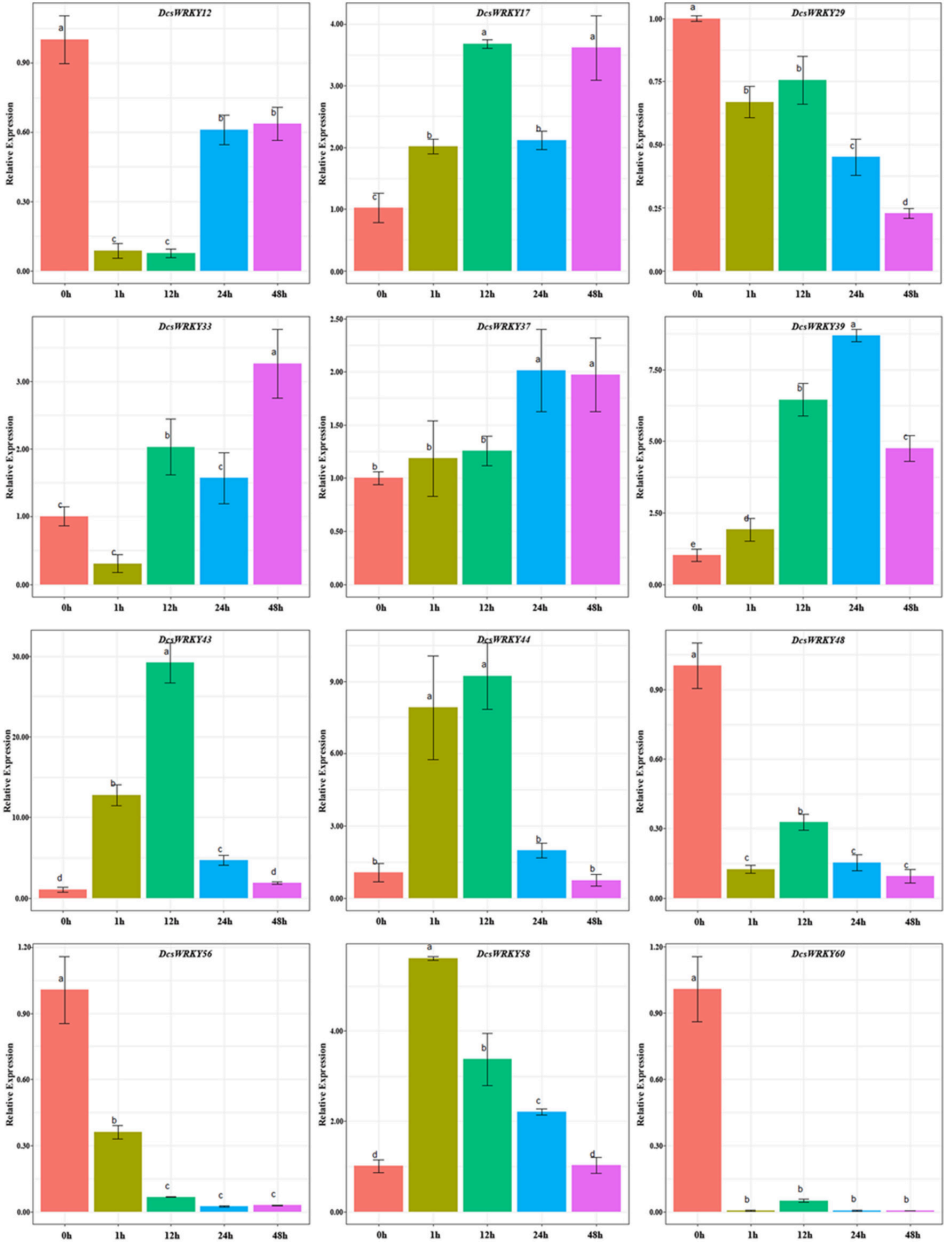
Expression patterns of *DcsWRKY* genes under GA treatments. Samples collected at 0, 1, 12, 24, and 48 h after treatment. The expression levels were normalized to control samples. Error bars represent standard errors of the mean in three biological replicates. One-way ANOVA test (*P* < 0.05, LSD) was used to statistically evaluate the significance among those samples. The histogram bars labeled with different letters (a, b, c, and d) above histogram bars are significantly different (LSD test, *P* < 0.05). LSD designates Fisher's least significant difference test.

GARE motif, TATC box, and p-box are gibberellin-response *cis*-acting elements (Rogers et al., 1994; Chen T. et al., 2012). In order to understand expression patterns of *DcsWRKY* genes with them, 12 genes were examined after leaves were sprayed with GA. In this study, GA treatment resulted in a wide variety of *DcsWRKY* gene expression profiles. As described in Figure 7, gene expression levels of *DcsWRKY12, DcsWRKY48, DcsWRKY56*, and *DcsWRKY60* decreased rapidly by 11, 8, 2.7, and 231 times after 1 h. However, the trend in expression of *DcsWRKY58* initially increased and peaked at 1 h, followed by a decrease. In addition, tandem duplicated genes (*DcsWRKY43* and *DcsWRKY44*) had a similar trend under GA treatment: there was an initial increase and reach a maximum level at 12 h, followed by a decrease.

**Figure 8:**
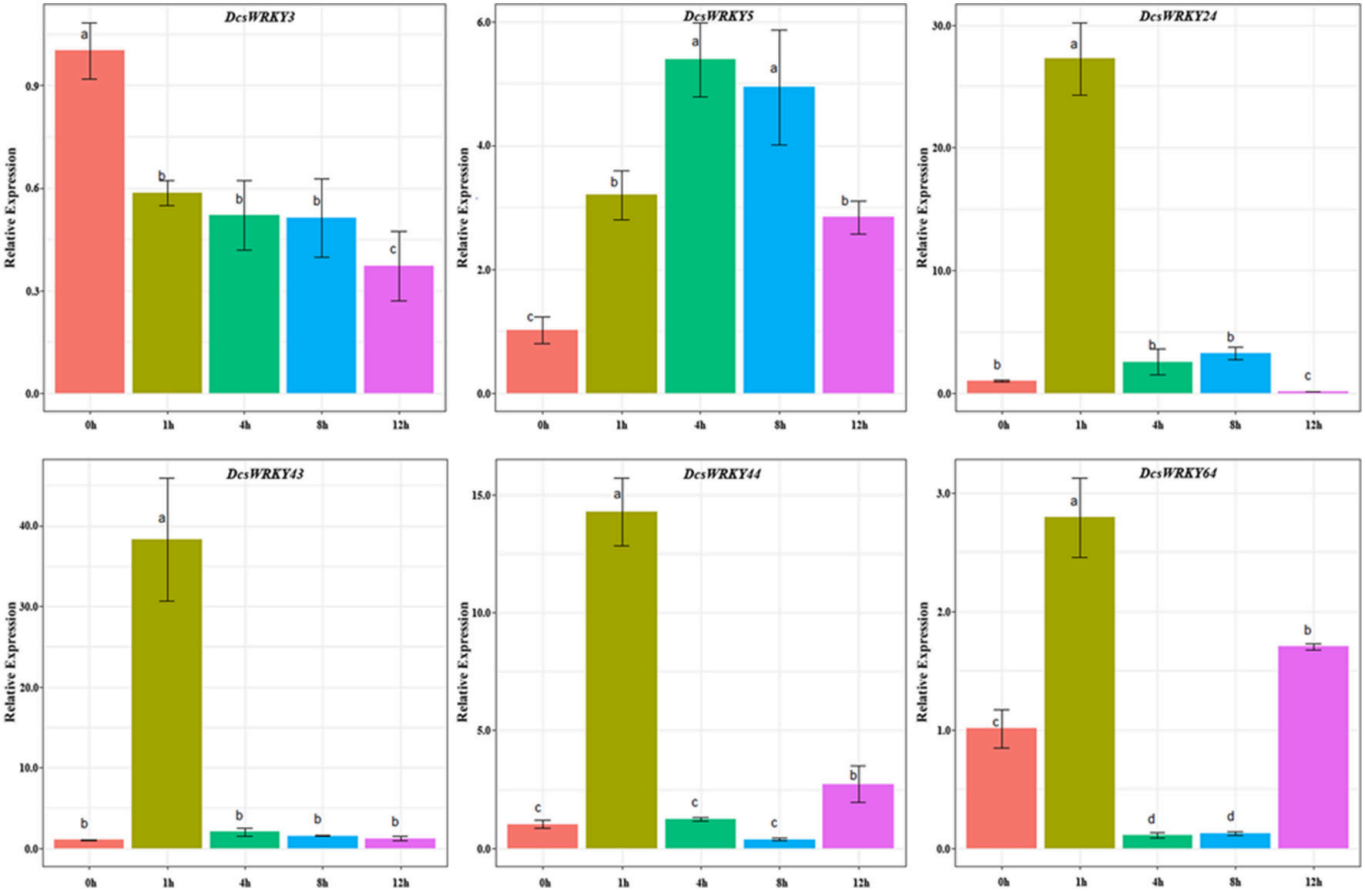
Expression patterns of *DcsWRKY* genes under mechanic injury treatments. Samples collected at 0, 1, 4, 8, and 12 h after treatment. The expression levels were normalized to control samples. Error bars represent standard errors of the mean in three biological replicates. One-way ANOVA test (*P* < 0.05, LSD) was used to statistically evaluate the significance among those samples. The histogram bars labeled with different letters (a, b, c, and d) above histogram bars are significantly different (LSD test, *P* < 0.05). LSD designates Fisher's least significant difference test.

The expression patterns of the six carrot *WRKY* genes with wound-responsive element (WUN-motif) were also investigated through qRT-PCR experiments. Our results showed that these *WRKY* genes were expressed in distinct behaviors. As shown in Figure 8, after 1 and 4 h treatment of mechanic damage, we observed sharp increase and decrease for some genes, such as *DcsWRKY24, DcsWRKY43, DcsWRKY44*, and *DcsWRKY64*. The expression levels of *DcsWRKY3* decreased rapidly in leaves after 1 h treatment and then decreased slightly, after 4, 8 and 12 h. The trend in the expression of *DcsWRKY5* initially increased and reached a maximum after 4 h and then decreased in 8 and 12 h. Moreover, *DcsWRKY43* and *DcsWRKY44* also showed a similar expression trend under mechanic injuries.

## Discussions

The *WRKY* gene family, one of the largest transcription factor families, is found to involve in a variety of important functions and have been extensively investigated in many plants (Zhou et al., 2016; Xiao et al., 2017; Yang et al., 2017). In this study, we accurately identified a total of 67 *WRKY* genes in the high-quality Nantes type carrot genome sequences, which are fewer than that (95 genes, Kuroda type carrot) previously reported in a draft genome assembly of *D*. *carota* L. cv. *Kuroda* (Xu et al., 2014). The chromosome-scale genome assembly has the advantage not only to characterize almost all members of the *WRKY* gene family but also localize their positions on chromosomes. We found that *DcsWRKY* genes are unevenly distributed on chromosomes. Chromosome two contained the majority of *DcsWRKY* genes (up to 17 of 67 *DcsWRKY* genes), while chromosome nine only had three. Our carrot genome collinearity analysis suggested that WGD events may have played a major role in driving the *DcsWRKY* gene family evolution, and all *DcsWRKY* gene pairs are under strong purifying selection. Previous study observed expression patterns of *DcWRKY* genes at different developmental stages of roots and under heat, cold, salt, and drought stresses (Li M. Y. et al., 2016). In this study, we examined tissue-specific expression profiling of *DcsWRKY* genes in different tissues and the abundance of *WRKY* genes under ABA, GA, and mechanic injury treatments. These efforts together shed new light on the evolution and functional divergence of the *WRKY* gene family in carrot.

Through a genome-wide analysis, a total of 67 *DcsWRKY* genes were identified in Nantes type carrot. And, our phylogenetic analysis demonstrated that *DcsWRKY*s could be divided into three major groups, among which group II was further classified into five subgroups, in accordance with the classification of *WRKY*s in *Arabidopsis*, rice, grape, tomato, and cucumber (Wu et al., 2005; Huang et al., 2009, 2012; Guo et al., 2014). Comparisons of conserved motifs in *DcsWRKY* members also supported the classification of *DcsWRKY* genes, indicating that the *WRKY* genes, to a certain extent, were functionally conserved among plants.

Compared with carrot (67 *DcsWRKY*s; genome size 473 Mb), a comparable number of *WRKY*s were identified in *Arabidopsis* (72; genome size 125 Mb), although the number of *WRKY*s was fewer in cucumber (55; genome size 367 Mb) and grape (59; genome size 487 Mb) and more in tomato (81; genome size 900 Mb) and rice (103; genome size 389 Mb) (International Rice Genome Sequencing, 2005; Jaillon et al., 2007; Huang et al., 2009, 2012; Ling et al., 2011; Tomato Genome, 2012; Guo et al., 2014; Iorizzo et al., 2016; Zhou et al., 2016). These results indicate that the number of *WRKY* genes may not be associated with the genome size. Moreover, the subgroup distribution of *WRKY* genes among plant species were also different. As showed in Figure 4, numbers of subgroups II-b, II-d, and II-e in carrot are larger than those of *A. thaliana* and grape, suggesting that carrot *WRKY*s of the three subgroups might have experienced linage-specific amplification. In this study, the collinearity analysis of *DcsWRKY* genes also support the conjecture and showed that more than half of the subgroup II-b, II-d, and II-e were undergo duplication events. Major *WRKY* members of subgroup II-b and II-e expand during Dc-α or Dc-β events, and most of subgroup II-d expand during γ paleohexaploidy event.

Gene duplication is an important driving force during plant evolution, which plays a central role in gene family expansion (Makova and Li, 2003; Li et al., 2005). Previous studies have demonstrated that gene duplication largely accounts for new gene functions (Ohno, 1970). In this study, we found 36 *DcsWRKY* genes were segmentally duplicated but only two genes were tandemly duplicated, indicating that segmental duplications make a great contribution to the expansion of *DcsWRKY* genes. Since tissue-specific expression patterns can provide clues for gene functional divergence during the evolution (Yao et al., 2015), we validated the expression of *DcsWRKY* genes in leaves, roots and stems through qRT-PCR. All tested 12 genes showed different expression levels, of which *DcsWRKY43* and *DcsWRKY44*, for example, were tandemly duplicated and showed a similar expression trend but *DcsWRKY43* was extremely highly expressed in stems. Moreover, *DcsWRKY29* and *DcsWRKY43* were WGD-derived duplicated gene pairs, which were expressed highly in leaves and lowly in stems, and lowly in leaves and highly in stems, respectively. Our results indicate that the expanded *WRKY* genes might result in novel biological function to remove their genetic redundancy and functional divergence might have occurred after gene duplication.

Previous studies founded that *WRKY* genes have complex regulatory networks in biotic and abiotic stresses and hormone responses (Chen L. et al., 2012; Phukan et al., 2016). In order to provide a foundation for further study of *WRKY* genes in carrot, the expression patterns of *DcsWRKY*s containing the corresponding stress signal *cis*-regulatory elements were evaluated at different time-points in response to ABA, GA as well as mechanic injury treatments. Consistent with previous studies (Guo et al., 2014; Wang et al., 2015), the expression levels of *WRKY* genes were rapidly induced by ABA and GA treatments within a few hours, indicating that these genes may play an important role in stress responses in carrot. ABA is a stress hormone and plays essential roles in plant responses to abiotic stresses (Agarwal et al., 2011; Chen L. et al., 2012). In our study, *DcsWRKY58* (II-a) was involved in the ABA signal transduction pathway which showing down-regulated after ABA treatment and reaching the lowest level after 48 h (Figure 6). Previous studies showed that *AtWRKY40* (homologous to *DcsWRKY58*) acts as a protein interacting with ABA receptors and is involved in biotic and abiotic stresses (Zou et al., 2007; Chen L. et al., 2012). This result could provide support for our findings that *DcsWRKY58* not only participates in ABA signal transduction pathway, but also responses to biotic and abiotic stresses. Li M. Y. et al. (2016) showed that *DcWRKY31* (II-a) in Kuroda type carrot whose homologous gene in *Arabidopsis* is also *AtWRKY40*, were found to be evidently up-regulated under drought, salt and pathogenic stresses. Thus, we can further indicate that *DcsWRKY58* in Nantes type carrot might was involved in drought, salt, and pathogenic stresses and regulated abiotic stress responses depending on ABA signaling pathway. Another example was *DcsWRKY5* (II-e) in this study and *DcWRKY18* (II-e) (Li M. Y. et al., 2016) which were both homologous to *AtWRKY22* (AT4G01250.1). Gibberellin (GA) also plays an important role in biotic stresses and plant disease resistance responses. *ABF1* (*AfWRKY1*) and *ABF2* (*AfWRKY2*) had been implicated in GA and ABA signaling and were involved in seed germination (Rushton et al., 2012). Rice *OsWRKY71* and *OsWRKY51* were found to act as regulators of ABA-inducible pathway and GA-repressible pathway in aleurone cell (Xie et al., 2006). Our study showed that *DcsWRKY* genes with GA gibberellin-response *cis*-acting elements had a variety of response patterns after GA treatments, indicating that they might be induced by hormones. Wounding is a common damage for plants and presents a constant threat to plants survival. It not only physically damages plant, but also provides pathways for pathogen invasion. However, there were only a few reports about transcriptional abundance of *WRKY* genes under wounding treatment. In *Arabidopsis, AtWRKY15, AtWRKY22*, and *AtWRKK33* were induced by wounding stress (Cheong et al., 2002). In this study, carrot homologs of these three *WRKY*s (*DcsWRKY43*/*DcsWRKY44, DcsWRKY5*, and *DcsWRKY64*) were also induced by wounding treatments, suggesting the potential functions of these *DcsWRKY* genes in mechanic injury stresses. Additionally, it was found that genes belonging to the same subgroup showed a distinct expression trend, such as *DcWRKY5* (II-e), *DcsWRKY18* (II-e), and *DcsWRKY27* (II-e) in ABA treatments, *DcWRKY17* (II-d), *DcsWRKY29* (II-d), and *DcsWRKY33* (II-d) in GA treatments. The different expression trends of *DcsWRKY* genes under abiotic stresses suggested that they might respond to abiotic stresses through different genetic networks. It has been reported that the co-expressed/co-responsive genes are likely to have common regulatory motifs in their promoters and are possibly regulated by a common set of TFs (Liu et al., 2005). *DcsWRKY43* and *DcsWRKY44* showed similar expression trend under GA and mechanic damage treatments and had the same *cis*-acting regulatory elements in upstream promoter regions, suggesting that these two genes may be regulated by a common set of TFs under stresses. Our expression analysis indicated that *DcsWRKY* genes were expressed in a tissue-specific behavior, some of which were in response to hormone signals, and mechanic injury stresses. This comprehensive analysis will enhance our understanding of the evolution and functional diversification of the *WRKY* gene.

## Author Contributions

HN performed data analysis, experiments, and drafted the manuscript. LG served as the principal investigator, facilitated the project, and revised the manuscript.

### Conflict of Interest Statement

The authors declare that the research was conducted in the absence of any commercial or financial relationships that could be construed as a potential conflict of interest.
